# Children's Hospital, Nottingham

**Published:** 1899-02-11

**Authors:** T. Fox Morrish


### EDITOR'S LETTER-BOX.

[Our Correspondents are reminded that prolixity is a great bar to publication, and that brevity of style and conciseness of statement greatly facilitate early insertion.]

### CHILDREN'S HOSPITAL, NOTTINGHAM.

MR. T. FOX MORRISH writes: May I ask you, in justice to my daughter and myself to insert this letter and accompanying statement in your next issue? The innuendo in the chairman's letter to yourself, that the complaint does not come from my daughter, is quite uncalled-for. In the very first letter which I wrote to his committee, dated October 13th, I said: "This matter is submitted from a deep feeling of professional and paternal moral responsibility." Again, on November 6th, I wrote: "As a father, I had a right to ask why my daughter, in violation of all your rules and time-table, was placed on duty day and night amid surroundings which I cannot but consider indecent and degrading." The statement of facts is my daughter's, but the complaint is my own, her father and guardian. With reference to the singular story of the baptism of the child, I can only say that those who know the Prayer-Book must look upon the explanation as very strange in the face of the Church's direction that parents are to be warned that without great cause and necessity they procure not their children to be baptised at home in their houses. That this matter may be placed clearly and distinctly before the public, I formulate the following as my complaint:—

1. That about the end of the trial month my daughter was placed upon isolation duty and relegated to a separate part of the building with a patient suffering from an infectious disease, and on this duty was kept for twelve consecutive days and nights, compelled to catch what sleep she could in the ward and to take her meals also on duty; the only time allowed being one hour in twenty-four for fresh air. I say that this was a flagrant breach of the published regulations of the institution, a gross violation of the rules and time table, unfair and cruel alike to nurse and patient, who have to submit without demur or remedy. It was a procedure also against every principle of a well-conducted hospital.

2. I maintain that to place a young and inexperienced girl in an isolated building, and in charge of a sick child, with no mode of communication with the rest of the establishment except by opening a window and ringing a hand bell, and to keep her there alone for hours at a time and all night, was a dangerous, cruel, and unwarrantable strain.

3. I complain that my daughter's surroundings were indecent and degrading. There was nothing but the doctor's basin and a sink to wash in, and only a bare table or washstand to eat off. Yet this is an institution which the management paraded as nursed entirely by gentlewomen.

For the rest I must leave the matter for the public to judge. I may repeat what I have already said to the management: "It is only by a knowledge of facts that a committee is prompted to make a thorough investigation into the internal administration of the institution under their control. If such a result ensues in the present instance the hospital may not only be made more Christ-like, but my daughter will not have suffered a grievous wrong in vain."

